# Sub-cellular markers highlight intracellular dynamics of membrane proteins in response to abiotic treatments in rice

**DOI:** 10.1186/s12284-018-0209-2

**Published:** 2018-04-12

**Authors:** Thi Thu Huyen Chu, Thi Giang Hoang, Duy Chi Trinh, Charlotte Bureau, Donaldo Meynard, Aurore Vernet, Mathieu Ingouff, Nang Vinh Do, Christophe Périn, Emmanuel Guiderdoni, Pascal Gantet, Christophe Maurel, Doan-Trung Luu

**Affiliations:** 1 BPMP, Univ Montpellier, CNRS, INRA, SupAgro, Montpellier, France; 2 National key laboratory for Plant Cell Biotechnology, Agricultural Genetics Institute, Pham Van Dong, Co Nhue, Tuliem, Hanoi, Vietnam; 3 CIRAD, UMR AGAP, 34398 Montpellier, France; 4 Université de Montpellier, CIRAD-INRA-Montpellier SupAgro, 34000 Montpellier, France; 5 University of Science and Technology of Hanoi, LMI RICE, 18 Hoang Quoc Viet, Nghia Do, Cau Giay, Hanoi, Vietnam; 6 IRD, LMI RICE, Agricultural Genetics Institute, Pham Van Dong road, Co Nhue, Tuliem, Hanoï, Vietnam; 7 Université de Montpellier, UMR DIADE, 911 Avenue Agropolis, 34394 Montpellier Cedex 5, France

**Keywords:** *Oryza sativa*, Subcellular markers, Intracellular dynamics, Abiotic stress

## Abstract

**Background:**

Cell biology approach using membrane protein markers tagged with fluorescent proteins highlights the dynamic behaviour of plant cell membranes, not only in the standard but also in changing environmental conditions. In the past, this strategy has been extensively developed in plant models such as Arabidopsis.

**Results:**

Here, we generated a set of transgenic lines expressing membrane protein markers to extend this approach to rice, one of the most cultivated crop in the world and an emerging plant model. Lines expressing individually eight membrane protein markers including five aquaporins (*Os*PIP1;1, *Os*PIP2;4, *Os*PIP2;5, *Os*TIP1;1, *Os*TIP2;2) and three endosomal trafficking proteins (*Os*Rab5a, *Os*GAP1, *Os*SCAMP1) were obtained. Importantly, we challenged in roots the aquaporin-expressing transgenic lines upon salt and osmotic stress and uncovered a highly dynamic behaviour of cell membrane.

**Conclusion:**

We have uncovered the relocalization and dynamics of plasma membrane aquaporins upon salt and osmotic stresses in rice. Importantly, our data support a model where relocalization of *Os*PIPs is concomitant with their high cycling dynamics.

**Electronic supplementary material:**

The online version of this article (10.1186/s12284-018-0209-2) contains supplementary material, which is available to authorized users.

## Background

In plants, popularization of cell biology approaches, such as laser scanning confocal microscopy, was promoted by the use in particular of shared sets of transgenic lines expressing fluorescent-protein fusions to subcellular markers. This approach has been mostly developed in the plant model Arabidopsis (Cutler et al. 2000). New sets of transgenic lines have also been developed in leading crop models such as maize (Krishnakumar et al. 2015), and rice (Wu et al. 2016). These sets of markers are interesting tools for highlighting subcellular compartments. Most interestingly, the use of protein markers with known biological functions allows one to uncover novel subcellular regulations. For instance, a set of multicolour markers of membrane compartments was used to study the intracellular dynamics of aquaporins in Arabidopsis (Wudick et al. 2015). Here, we present a new set of transgenic rice lines stably-expressing individually fluorescent protein fusions with subcellular protein-markers. These include (i) plasma membrane (PM) and tonoplast aquaporins (Sakurai et al. 2005), (ii) PM-marker low-temperature inducible protein 6A (LTi6a; (Cutler et al. 2000)), (iii) *Os*Rab5a known to be localized in a pre-vacuolar compartment (Shen et al. 2011), (iv) *Os*GAP1 which has a putative function for Golgi apparatus to PM and *trans*-Golgi network (TGN) trafficking and potentially localizes in endosomal compartments (Heo et al. 2005), and (v) a rice secretory carrier membrane protein (*Os*SCAMP1) which is localized in an early endosome compartment and may have a function in the early stage of membrane trafficking from the PM (Cai et al. 2011). Apart from aquaporins and LTi6a, all of these proteins are identified components of key compartments en route towards the vacuole. Importantly, we challenged the aquaporin-expressing transgenic lines upon salt and osmotic stress to uncover their dynamic behaviour in rice roots.

## Results and Discussion

### Rice Transgenic Line Creation and Subcellular Localization Visualization

**Fig. 1:**
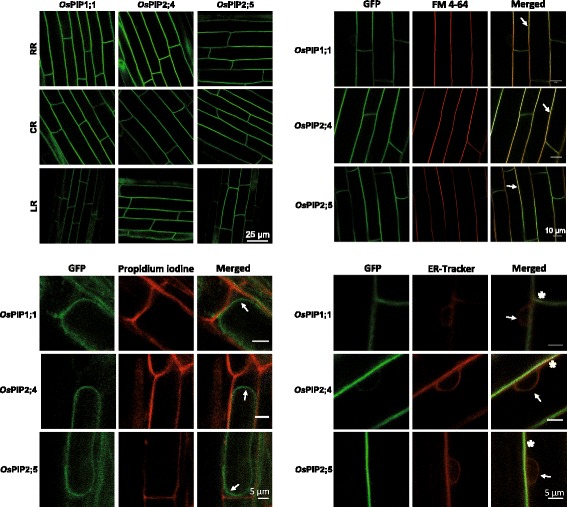
Subcellular localization of PM protein markers tagged with fluorescent proteins in rice root. Labelling of *Os*PIP-GFP constructions was observed in exodermis of fresh roots in different types (upper left). Each row of photos refers to distinct types: RR, radicle root; CR, crown root; LR, lateral root. Root cells were briefly stained with FM4–64, a typical PM-dye in these conditions, and showed a colocalization with *Os*PIP-GFP signal (arrows, upper right). Plasmolysis of protoplasts was observed with propidium iodide counterstaining the cell wall, and did not show any colocalization with *Os*PIP-GFP signal (arrows, lower left). ER-Tracker Blue-White DPX stained a compartment surrounding the nucleus, tentatively identified as endoplasmic reticulum (arrows, lower right). Due to limited resolution of light microscopy, PM and endoplasmic reticulum labelling could not be separated properly (asterisks). Root cells were observed by means of laser scanning confocal microscopy. Images were taken at a region ~ 0.5–1 cm from the root tip of plants, 7–8 days after germination

**Fig. 2:**
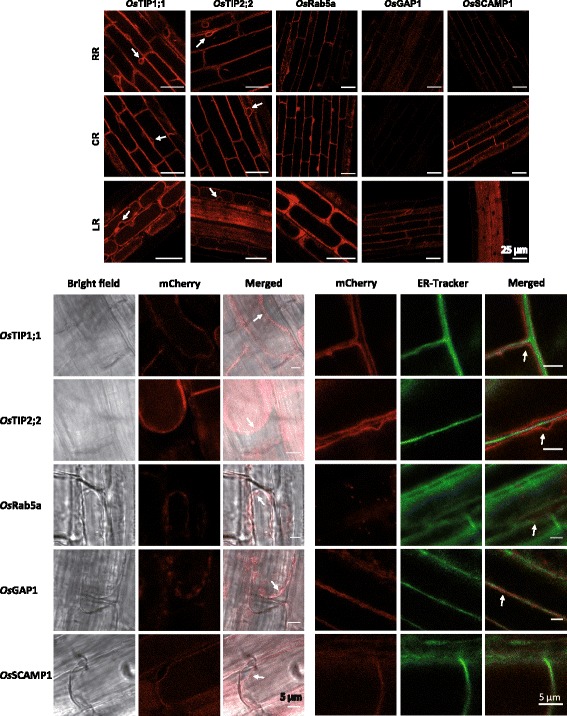
Subcellular localization of intracellular protein markers tagged with fluorescent proteins in rice root. Labelling of mCherry-construction (*Os*TIP1;1, *Os*TIP2;2, *Os*Rab5a, *Os*GAP1 and *Os*SCAMP1) was observed in exodermis of fresh roots in different types (upper). Note labelling of *Os*TIP-mCherry constructs that skirted the nuclei (arrows). Plasmolysis of protoplasts revealed a space between the labelling and the cell wall (arrows, lower left). ER-Tracker Blue-White DPX staining revealed in most of the case no colocalization with mCherry-constructs (arrows, lower right)

**Fig. 3:**
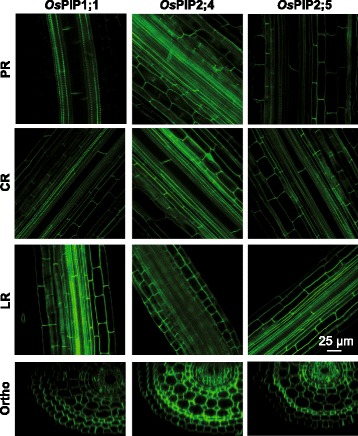
In depth imaging of *Os*PIPs in rice root. ClearSee technique was applied prior to observation of *Os*PIP-GFP labelling in deep root tissues by multiphoton excitation microscopy. Root types: RR, radicle root; CR, crown root; LR, lateral root. Ortho stands for orthogonal section after reconstitution of Z-stack images

The genes of interest were cloned in fusion with the sequence of a fluorescent protein and under the transcriptional control of a constitutive promoter. Rice PM aquaporin (*OsPIP1;1, OsPIP2;4, OsPIP2;5*) sequences were fused with the green fluorescent protein (*GFP*) sequence to form *OsPIP-GFP* constructs, and PM *LTi6a* was fused with the cyan fluorescent protein (*CFP*) to form a *CFP*-*LTi6a* construct. The other proteins were fused with the *mCherry* sequence. The expression cassettes were cloned in the binary vector pGreenII 0179 (Hellens et al. 2000) and transferred into either rice (*Oryza sativa* L. cv. Nipponbare) or *Arabidopsis thaliana* (Col-0 accession) by *Agrobacterium*-mediated transformation. When expressed in Arabidopsis, rice PM-aquaporin constructs were found to be located at their expected subcellular localization (Additional file 1: Figure S1). The anatomical organization of rice roots is more complex than in Arabidopsis, as it comprises in particular more cell layers (Rebouillat et al. 2009). In the present work, only the epidermis, exodermis, sclerenchyma and very first mesodermal cells could be visualized by confocal microscopy of rice root systems, and very weak autofluorescence background was detected there (Additional file 1: Figure S2). The fluorescent signal of *Os*PIPs in epidermis was weak. In contrast, the small and flat sclerenchyma cells, the exodermal and mesodermal cells exhibited a strong and homogeneous signal, amenable for confocal microscopy. When expressed in rice, *Os*PIP constructs showed a typical homogeneous labelling of the PM which colocalized with the fluorescent styryl dye FM4–64 (Fig. 1). To make sure of the absence of cell wall labelling, protoplasts were plasmolysed and observed with cell wall counterstained with propidium iodide (Fig. 1). Labelling of the endoplasmic reticulum around the nuclei with ER-Tracker Blue-White DPX revealed almost no labelling of this intracellular compartment by *Os*PIP-GFP constructs (Fig. 1). This series of clues supported the *bona fide* assumption of PM labelling by *Os*PIP-constructs. Tonoplast aquaporin (*Os*TIP-mCherry) constructs were associated with a labelling of intracellular invaginations that skirted the nuclei and are typical of the vacuolar membrane (Fig. 2). In addition, we could observe a consistent labelling of intracellular compartments by using the *Os*Rab5a, *Os*GAP1 and *Os*SCAMP1 markers (Fig. 2). Plasmolysis of the protoplasts revealed no labelling of the cell wall, while staining with ER-Tracker Blue-White DPX revealed in almost all mCherry-constructs absence of co-localization with the endoplasmic reticulum (Fig. 2). The thickness of rice roots is a limitation to the observation of deep tissues by laser scanning confocal microscopy. We overcome this limitation by means of the ClearSee technique which was initially developed to image in depth the morphology and gene expression of plant tissues (Kurihara et al. 2015). This technique has a lot of advantages such as diminishing chlorophyll auto-fluorescence while preserving fluorescent protein stability. It is applicable to multicolour imaging of fluorescent proteins and compatible with chemical staining. It also allows long-term storage of sampled tissues. In addition, multiphoton excitation microscopy (MPEM) can provide a deeper penetration of infra-red light into plant tissues (Feijo and Moreno 2004). Though the overall autofluorescence background is weak, caution should be taken since a higher signal is detected in the xylem vessels (Additional file 1: Figure S2). By combining ClearSee and MPEM, the PM signal of *Os*PIP-GFP constructs could be visualized down to the central cylinder at a depth of ~ 150 μm (Fig. 3). Although use of protein markers tagged with fluorescent protein should be done with caution, since sometimes the fusion could affect the subcellular localization, several reports addressed biological questions with this strategy. We have established here a new set of transgenic rice plants enlightening multiple cell compartments.

#### Redistribution of PM Aquaporins upon Salt and Drought Stress

**Fig. 4:**
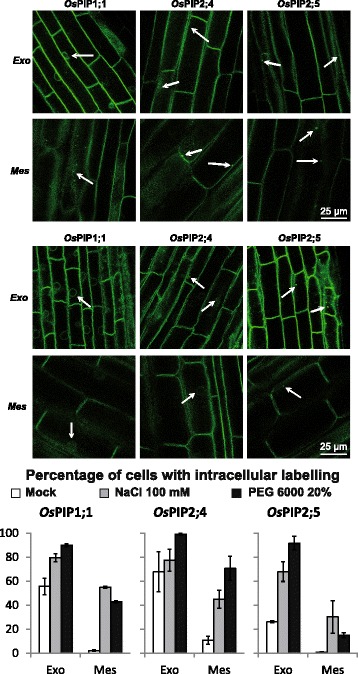
Effects of salinity and osmotic stress on subcellular localization of rice PM aquaporins in root. Control, salt or PEG treatments were applied on whole root systems and crown root cells were observed by laser scanning confocal microscopy. Intracellular labelling observed upon salt (upper) and osmotic stress (middle) are indicated with arrows. Each column refers to an isoform and each line refers to a cell layer, either exodermis (Exo) or mesodermis (Mes). (Lower) Quantification of intracellular labelling upon either control treatment with water (opened bars), 100 mM NaCl (grey bars) or 20% (*w*/*v*) PEG6000 (closed bars) for 30 min, respectively. The mean values and ± SEs are indicated

This set of markers was also used for a dynamic survey of membrane compartments upon environmental challenges. To investigate the behaviours of aquaporins upon salt and osmotic stress, roots of transgenic lines expressing either *Os*PIP1;1*, Os*PIP2;4 or *Os*PIP2;5 constructs were challenged with 100 mM NaCl or 20% (*w*/*v*) PEG6000 for 30 min and observed by confocal microscopy. Firstly, we validated the behaviour of the *Os*PIP constructs in Arabidopsis and observed a specific relocalization of these PM aquaporins into intracellular compartments depending on the isoform, the cell type or the treatment (Additional file 1: Figure S3). For instance, *Os*PIP2 isoforms exhibited a stronger tendency to relocalize upon abiotic stress than *Os*PIP1;1. Secondly, when expressed in rice crown root cells, the *Os*PIP constructs exhibited a marked intracellular labelling (Fig. 4). In any case, both the NaCl and PEG stresses resulted in a marked increase of intracellular labelling as compared to control conditions. For instance, in exodermal cells, we noticed that *Os*PIP1;1 construct labelled a compartment surrounding the nucleus, tentatively identified as the endoplasmic reticulum. Redistributions of *Os*PIP2;4 and *Os*PIP2;5 were observed mainly in punctuated structures. In Arabidopsis, upon drought stress, a RING membrane-anchor E3 ubiquitin ligase has been reported to be involved in the ubiquitination of *At*PIP2;1 and the retention in the endoplasmic reticulum of this aquaporin (Lee et al. 2009). In mesodermal cells, intracellular labelling with the *Os*PIP1;1 construct was punctuated and detected in only 2% cells in control condition but in ~ 55% and 43% of cells, under salt and osmotic stress, respectively. The differences of subcellular redistribution of *Os*PIP1;1 and *Os*PIP2s, upon stress, between exodermal and mesodermal cells suggested an isoform-specific and cell-specific response. We observed a similar phenotype in mesodermal cells of radicle root (Additional file 1: Figure S4). The CFP-LTi6a construct showed a much lower tendency to relocalize in intracellular compartments upon salt or osmotic stress (Additional file 1: Figure S5). Importantly, a specificity of *Os*PIP relocalization upon these stresses was observed in rice, whereas such phenomenon was not reported for *At*PIP in Arabidopsis (Boursiac et al. 2008). Following the description of *At*PIP internalization upon salt and oxidative stress in Arabidopsis roots (Boursiac et al. 2008), the present work extends this behaviour to their orthologues in rice. Since this phenotype can be observed in two representative dicot and monocot species, we propose that it represents a conserved adaptive mechanism upon abiotic environmental stress.

#### Dynamic of Endocytosis of PM Aquaporins upon Salt Stress

**Fig. 5:**
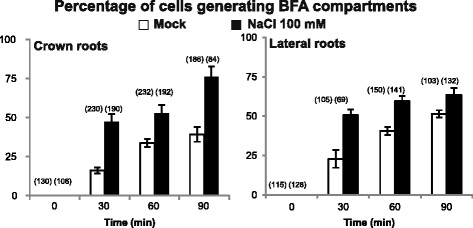
Effects of salt stress on the dynamics of brefeldin A (BFA) compartment labelling by *Os*PIP2;5 constructs in rice root cells. Root systems of *Os*PIP2;5-GFP expressing line were incubated in either water (mock condition; open bars) or 100 mM NaCl (closed bars), both supplemented with 50 μM BFA. The number of cells with at least one labelled BFA compartment was counted from images acquired by confocal microscopy, from time 0 to the indicated times. This number was then normalized to the total cell number observed of each root type (crown and lateral roots). Numbers in parentheses indicate the number of cells observed in each assay. The mean values and ± SEs are indicated

Brefeldin A (BFA) is a lactone antibiotic produced by fungal organisms which disturbs exocytosis by inhibiting the function of ADP-ribosylation factor-guanine-exchange factor. BFA thereby provokes the aggregation of endosomal vesicles including *trans*-Golgi network (TGN) compartment and induces the formation of large structures named as BFA compartments. We used *Os*PIP2;5 as a prototypic PM aquaporin together with BFA treatments to address the membrane protein cycling dynamics in rice, as investigated earlier in Arabidopsis (Luu et al. 2012). When de novo protein synthesis is prevented by a cycloheximide treatment (Jásik and Schmelzer 2014), the kinetics of BFA compartment labelling by PM markers can be used to probe the dynamics of endocytosis. Rice roots were exposed to BFA (50 μM) dissolved in either water or a 100 mM NaCl solution and corresponding to control or salt-stress conditions, respectively. In control conditions, *Os*PIP2;5-GFP labelled intracellular structures typical of BFA compartments, indicating that *Os*PIP2;5 traffics through the TGN compartment. After a 90 min BFA treatment, ~ 39% and 51% of exodermal cells exhibited BFA compartments of 2 μm and 1 μm in diameter, in crown roots and lateral roots, respectively (Fig. 5). In salt-stress conditions, we observed a higher percentage of root cells with a BFA compartment than in control conditions. For instance, exodermal cells of crown roots subjected for 30 min to a control or salt stress treatment showed ~ 16% and 47% of cells with a BFA compartment, respectively. These results suggest that salt treatment enhanced the endocytosis of *Os*PIP2;5. A similar result on *At*PIP isoforms was obtained in Arabidopsis (Luu et al. 2012). Effects of BFA might be concentration-dependent (Jásik and Schmelzer 2014; Lam et al. 2009). Therefore, we tested other concentrations (~ 100 μM) and found similar results (data not shown).

In conclusion, we have uncovered the relocalization and dynamics of PM aquaporins upon salt and osmotic stresses in rice. Importantly, our data support a model where relocalization of *Os*PIPs is concomitant with their high cycling dynamics. Altogether these data indicate that the rice research community has at its disposal a new set of subcellular markers amenable for cell biology approaches on a large array of topics.

## Methods

### Molecular Cloning of Membrane Protein Markers and Plant Transformation

Molecular cloning information is summarized in Additional file 2: Table S1. *OsPIP1;1* was subcloned into pDONR207 and transferred into the destination vector pGWB5 (Nakagawa et al. 2007) using Gateway® Gene Cloning technology (Invitrogen, USA), according to the manufacturer’s instruction. The whole set of the other protein markers were subcloned into pBluescript SK vector (Stratagene, USA) or pUC57 (see Additional file 2: Table S1), and then cloned into the pGreenII 0179 binary vector (Hellens et al. 2000) under the transcriptional and translational control of a double enhanced cauliflower mosaic virus *35S* promoter and the 3′ end of the pea ribulose-1,5-bisphosphate carboxylase small subunit *rbcS* gene. A molecular construct consisting of the maize ubiquitin-1 promoter controlling the expression of a fluorescent plasma-membrane-localized fusion protein (ECFP-LTI6a) (Cutler et al. 2000) was obtained by synthesis (Genscript) and cloned into the plasmid pUC57. The insert was released by a double digestion with *Eco*RI et *Kpn*I and cloned into the binary vector pCAMBIA2300 linearized by *Eco*RI and *Kpn*I. All constructs were confirmed by DNA sequencing by Eurofins Genomics (Germany). The recombinant DNA plasmids were electroporated into *Agrobacterium tumefaciens* strain EHA105 or GV3101 for rice or Arabidopsis transformation, respectively. *Japonica* rice Nipponbare cultivar was transformed according to a modified seed-embryo callus transformation procedure (Sallaud et al. 2003). Arabidopsis transformation was performed according to flower dip protocol (Clough and Bent 1998). Selection of transgenic plants was performed with medium supplemented with hygromycin.

### Plant Materials and Growth Conditions

Rice *Oryza sativa* L. cv. Nipponbare and *Arabidopsis thaliana* L. (Heyn.) accession Columbia 0 were used in this study. Rice seeds were dehusked, then sterilized by dipping in 70% ethanol for 2 min, soaking in 3.6% (*w*/*v*) sodium hypochlorite solution for 30 min and rinsing several times with distilled water. After sterilization, the seeds were put in the petri dish containing moist filter paper. After emergence of the coleoptile and germination of the radicle, seeds were transferred onto a raft floating on deionised water. Conditions of the growth chamber were 14 h of day cycle (~ 200 μE m^− 2^ s ^− 1^) and 10 h of night at 28/25 °C and 70% relative humidity. Seven to eight days after germination (DAG) rice seedlings were used for cell biology approaches. Arabidopsis seeds were surface sterilized in a solution (50% ethanol, 4 g L^− 1^ Bayrochlore and 0.02% (*w/v*) Clean N for 10 min, thoroughly washed with 70% ethanol and air-dried under the sterile hood for 2 h. Sterilized seeds were sown on sucrose-added (10 g L^− 1^) half-strength Murashige and Skoog (MS) medium (Murashige and Skoog 1962) in clear polystyrene plates (12 × 12 cm) sealed with an air-permeable tape. After 48 h of stratification in 4 °C dark room, plates were transferred vertically into a growth chamber with cycles of 16 h of light (~ 150 μE m^− 2^ s ^− 1^) and 8 h of dark at 21 °C and 65% relative humidity. Seven days after sowing (DAS), Arabidopsis seedlings were used for cell biology approaches.

### Confocal Microscopy Visualization

A laser scanning confocal microscope (Leica TSC SP8 system, Germany) was used with the excitation wavelengths 405 nm, 488 nm and 561 nm for CFP, GFP and mCherry, respectively. The detection wavelengths were in the range of 450–500 nm for CFP, 500–535 nm for GFP and 580–630 nm for mCherry. For ER-Tracker Blue-White DPX staining, observations were made with excitation wavelength 405 nm and detection wavelengths in the range of 420–500 nm. For FM4–64 and propidium iodide staining, observations were made with excitation wavelength 561 nm and detection wavelengths in the range of 580–630 nm. Images were taken at a region ~ 0.5–1 cm from the root tips. Images were captured in a z-stack of 0.5 μm intervals for subcellular localization and a time lapse for mobility of protein observation.

Multiphoton microscope (Zeiss LSM 7MP OPO, Germany) was used to observe ClearSee-prepared tissue with the excitation wavelength 836 nm and signals were collected in detection range of 500–550 nm.

### ClearSee Tissue Preparation

ClearSee solution was prepared by mixing xylitol (10% *w/v*), sodium deoxycholate (15% *w/v*) and urea (25% *w/v*) in water (Kurihara et al. 2015). Briefly, rice seeds were dehusked and surface sterilized in 3.6% (*w*/*v*) sodium hypochlorite solution for 30 min, then washed carefully with sterile water. Next, seeds were sown on half-strength MS medium in clear polystyrene plates (24.5 × 24.5 cm), 25 seeds for 1 line at the middle per plate. Plates were kept vertically in culture room at 29 °C for 12 h of day, 25 °C for 12 h of night and relative humidity at 66%. Seven DAG, rice roots were collected and immediately immersed in a 4% (*w/v*) paraformaldehyde solution prepared with 1X PBS, subjected to vacuum for 30 min, washed again with 1X PBS, and then immersed in ClearSee solution under vacuum for 2 h, followed by 1 week at ambient conditions. When rice root became transparent, each root type was mounted in a 1% (*w/v*) agarose solution and visualized by means of a multiphoton microscope.

### Fluorescent Dye Staining

We used 1 μM ER-Tracker Blue-White DPX (1 mM stock solution in dimethylsulfoxide), 10 μM FM4–64 (10 mM stock solution in H_2_O), and propidium iodide 1 μg/mL (1 mg/mL stock solution in water). Dyes were from Molecular Probes (Eugene, USA) and diluted into water before root staining. Roots were immersed into FM4–64 for 2–5 min before observation. For propidium iodide staining, roots were stained for 5 min, and then transferred into a 500 mM mannitol solution for plasmolysis before observation. Staining with ER-Tracker Blue-White DPX was carried out for 30–60 min before observation.

### Stress Application and Pharmacological Approach

Plants were stress challenged by incubating the roots in solutions of 100 mM NaCl or 20% (*w/v*) PEG6000. Brefeldine A was used at a concentration of 50 μM, dissolved into water supplemented or not with 100 mM NaCl. Importantly, cycloheximide was added at a concentration of 50 μM prior to and during BFA treatments to prevent new protein biosynthesis. All chemicals listed in this section were from Sigma-Aldrich (USA).

## Additional files

Additional file 1: **Figure S1.** Subcellular localisation of rice proteins in Arabidopsis root cells. **Figure S2.** Autofluorescence background in rice primary root cells. **Figure S3.** Re-localization of rice aquaporins in Arabidopsis root under salt and osmotic stresses. **Figure S4.** Re-localization of plasma membrane aquaporins in rice primary root under salt and osmotic stresses. **Figure S5.** Subcellular localization of plasma-membrane protein-marker LTi6a-CFP in rice root under salt and osmotic stresses. (PDF 623 kb)

Additional file 2: **Table S1.** Overview of molecular cloning of membrane protein markers. (DOCX 16 kb)

## References

[CR1] Boursiac Y, Boudet J, Postaire O, Luu D-T, Tournaire-Roux C, Maurel C (2008). Stimulus-induced downregulation of root water transport involves reactive oxygen species-activated cell signalling and plasma membrane intrinsic protein internalization. Plant J.

[CR2] Cai Y, Jia T, Lam SK, Ding Y, Gao C, San MW, Pimpl P, Jiang L (2011). Multiple cytosolic and transmembrane determinants are required for the trafficking of SCAMP1 via an ER-Golgi-TGN-PM pathway. Plant J.

[CR3] Clough SJ, Bent AF (1998). Floral dip: a simplified method for agrobacterium-mediated transformation of Arabidopsis thaliana. Plant J.

[CR4] Cutler S, Ehrhardt D, Griffitts J, Somerville C (2000). Random GFP::cDNA fusions enable visualisation of subcellular structures in cells of Arabidopsis at a high frequency. Proc Natl Acad Sci U S A.

[CR5] Feijo J, Moreno N (2004). Imaging plant cells by two-photon excitation. Protoplasma.

[CR6] Hellens RP, Edwards EA, Leyland NR, Bean S, Mullineaux PM (2000). pGreen: a versatile and flexible binary Ti vector for agrobacterium-mediated plant transformation. Plant Mol Biol.

[CR7] Heo JB, Rho HS, Kim SW, Hwang SM, Kwon HJ, Nahm MY, Bang WY, Bahk JD (2005). OsGAP1 functions as a positive regulator of OsRab11-mediated TGN to PM or vacuole trafficking. Plant Cell Physiol.

[CR8] Jásik J, Schmelzer E (2014). Internalized and newly synthesized Arabidopsis PIN-FORMED2 pass through brefeldin a compartments: a new insight into intracellular dynamics of the protein by using the photoconvertible fluorescence protein Dendra2 as a tag. Mol Plant.

[CR9] Krishnakumar V, Choi Y, Beck E, Wu Q, Luo A, Sylvester A, Jackson D, Chan AP (2015). A maize database resource that captures tissue-specific and subcellular-localized gene expression, via fluorescent tags and confocal imaging (maize cell genomics database). Plant Cell Physiol.

[CR10] Kurihara D, Mizuta Y, Sato Y, Higashiyama T (2015). ClearSee: a rapid optical clearing reagent for whole-plant fluorescence imaging. Development.

[CR11] Lam SK, Cai Y, Tse YC, Wang J, Law AHY, Pimpl P, Chan HYE, Xia J, Jiang L (2009). BFA-induced compartments from the Golgi apparatus and trans-Golgi network/early endosome are distinct in plant cells. Plant J.

[CR12] Lee HK, Cho SK, Son O, Xu Z, Hwang I, Kim WT (2009). Drought stress-induced Rma1H1, a RING membrane-anchor E3 ubiquitin ligase homolog, regulates aquaporin levels via ubiquitination in transgenic Arabidopsis plants. Plant Cell.

[CR13] Luu D-T, Martinière A, Sorieul M, Runions J, Maurel C (2012). Fluorescence recovery after photobleaching reveals high cycling dynamics of plasma membrane aquaporins in Arabidopsis roots under salt stress. Plant J.

[CR14] Murashige T, Skoog F (1962). A revised medium for rapid growth and bioassays with tobacco tissue cultures. Physiol Plant.

[CR15] Nakagawa T, Kurose T, Hino T, Tanaka K, Kawamukai M, Niwa Y, Toyooka K, Matsuoka K, Jinbo T, Kimura T (2007). Development of series of gateway binary vectors, pGWBs, for realizing efficient construction of fusion genes for plant transformation. J Biosci Bioeng.

[CR16] Rebouillat J, Dievart A, Verdeil JL, Escoute J, Giese G, Breitler JB, Gantet P, Espeout S, Guiderdoni E, Perin E (2009). Molecular genetics of rice root development. Rice.

[CR17] Sakurai J, Ishikawa F, Yamaguchi T, Uemura M, Maeshima M (2005). Identification of 33 rice aquaporin genes and analysis of their expression and function. Plant Cell Physiol.

[CR18] Sallaud C, Meynard D, van Boxtel J, Gay C, Bes M, Brizard JP, Larmande P, Ortega D, Raynal M, Portefaix M, Ouwerkerk PB, Rueb S, Delseny M, Guiderdoni E (2003). Highly efficient production and characterization of T-DNA plants for rice ( Oryza sativa L.) functional genomics. Theor Appl Genet.

[CR19] Shen Y, Wang J, Yu D, Lo SW, Gouzerh G, Neuhaus J-M, Jiang L (2011). The Rice RMR1 associates with a distinct prevacuolar compartment for the protein storage vacuole pathway. Mol Plant.

[CR20] Wu T-M, Lin K-C, Liau W-S, Chao Y-Y, Yang L-H, Chen S-Y, Lu C-A, Hong C-Y (2016). A set of GFP-based organelle marker lines combined with DsRed-based gateway vectors for subcellular localization study in rice (Oryza sativa L.). Plant Mol Biol.

[CR21] Wudick MM, Li X, Valentini V, Geldner N, Chory J, Lin J, Maurel C, Luu D-T (2015). Subcellular redistribution of root aquaporins induced by hydrogen peroxide. Mol Plant.

