# Supplementary material for: Sub-cellular markers highlight intracellular dynamics of membrane proteins in response to abiotic treatments in rice
Source: Rice (N Y). 2018 Apr 12;11:23. doi: 10.1186/s12284-018-0209-2 (PMC5897272; doi:10.1186/s12284-018-0209-2)
Supplement: Supplementary file 1 — Figure S1. Subcellular localisation of rice proteins in Arabidopsis root cells. Figure S2. Autofluorescence background in rice primary root cells. Figure S3. Re-localization of rice aquaporins in Arabidopsis root under salt and osmotic stresses. Figure S4. Re-localization of plasma membrane aquaporins in rice primary root under salt and osmotic stresses. Figure S5. Subcellular localization of plasma-membrane protein-marker LTi6a-CFP in rice root under salt and osmotic stresses. (PDF 623 kb) [file 12284_2018_209_MOESM1_ESM.pdf]

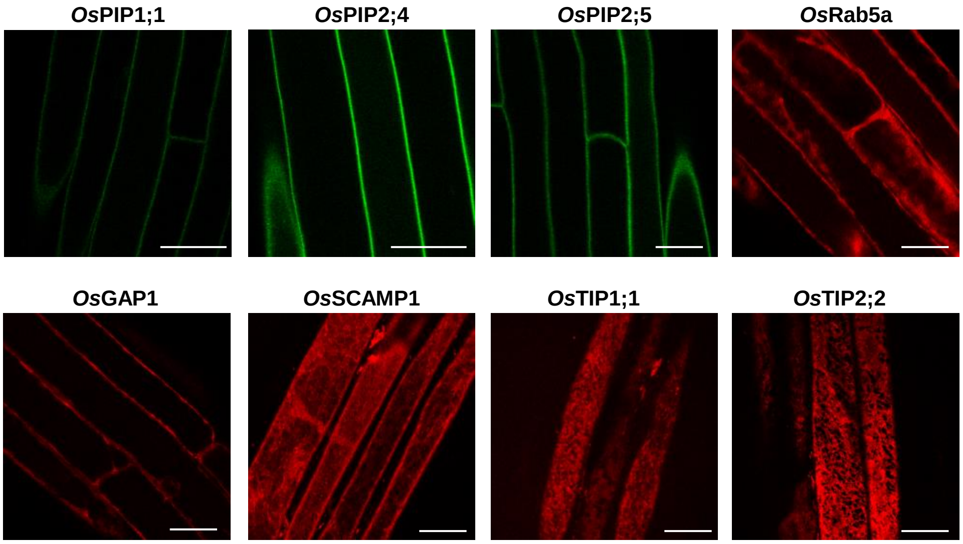

Supplementary Figure S1. Subcellular localisation of rice proteins in Arabidopsis root cells.
Fresh roots in half-strength MS medium were observed by laser scanning confocal microscopy (LSCM). In Arabidopsis, *Os*PIPs localized in the PM but *Os*TIPs and *Os*SCAMP1 were slightly retained in endoplasmic reticulum, other markers distributed as expected in cytoplasm. Scale bar = 20 µm.

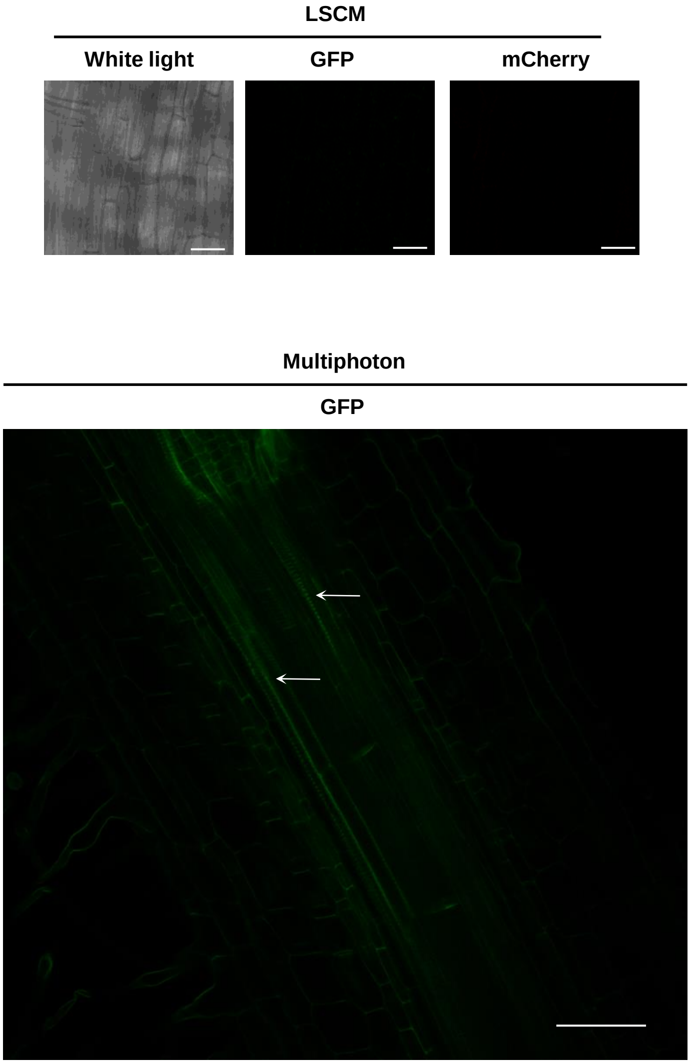

Supplementary Figure S2. Autofluorescence background in rice primary root cells.
(Upper) Fresh root of non-transgenic rice was observed by LSCM using either white light, GFP, or mCherry channel. (Lower) Root system of non-transgenic rice was subjected to ClearSee technique and observed by means of mutiphoton excitation microscopy; weak autofluorescence background was detected, especially in xylem vessels (arrows). Scale bar = 25 µm.

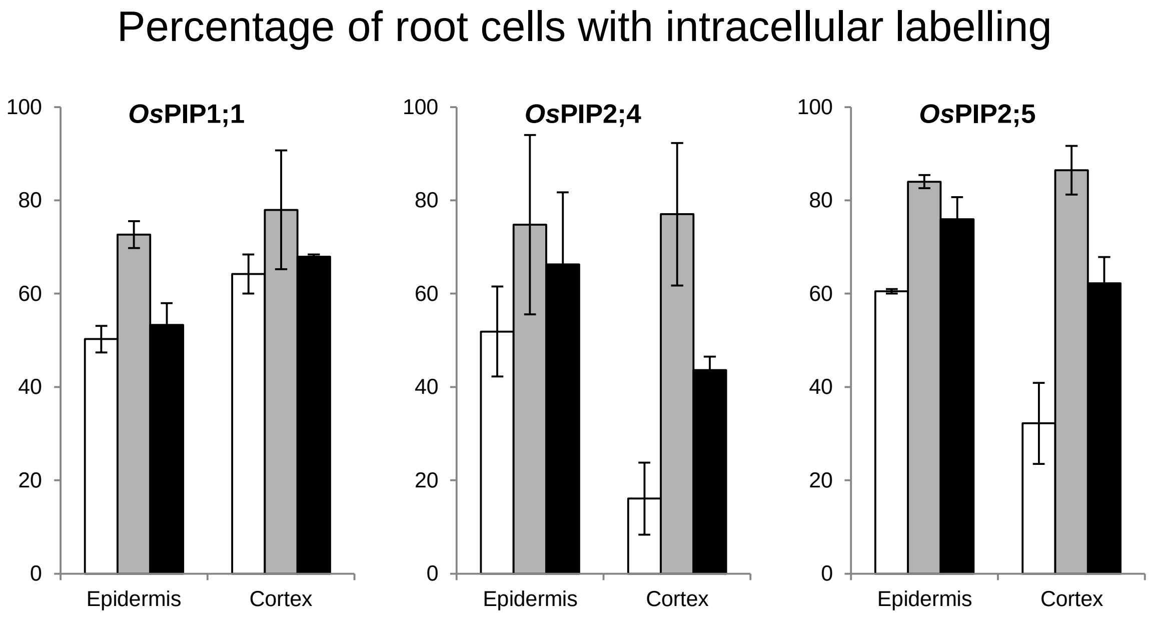

Supplementary Figure S3. Re-localization of rice aquaporins in Arabidopsis root under salt and osmotic stresses

Charts show the percentage of root cells displaying intracellular labelling in either epidermis or cortex. Open, grey and closed bars correspond to mock (half-strength MS liquid medium), 100 mM NaCl and 20% (w/v) PEG6000, respectively. Experiments were done 2 times for each construct, ~10 plants were observed for each experiment, ~6 cells were analysed for each plant. The mean values and SEMs are indicated.

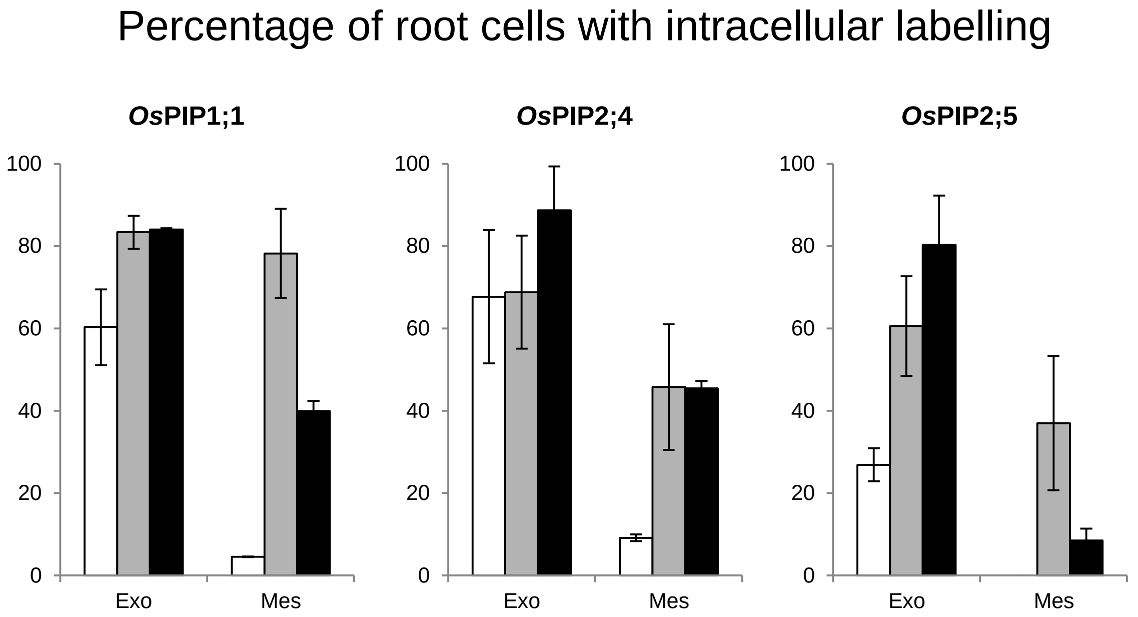

Supplementary Figure S4. Re-localization of plasma membrane aquaporins in rice primary root under salt and osmotic stresses

Charts show the percentage of root cells displaying intracellular labelling in either exodermis (Exo) or mesodermis (Mes). Open, grey and closed bars correspond to mock (half-strength MS liquid medium), 100 mM NaCl and 20% (w/v) PEG6000, respectively. *Os*PIP-GFP constructs were observed in primary roots. Experiments were done 2 times for each construct, 4-6 plants were observed for each experiment, ~10 cells were analysed for each plant. The mean values and SEMs are indicated.

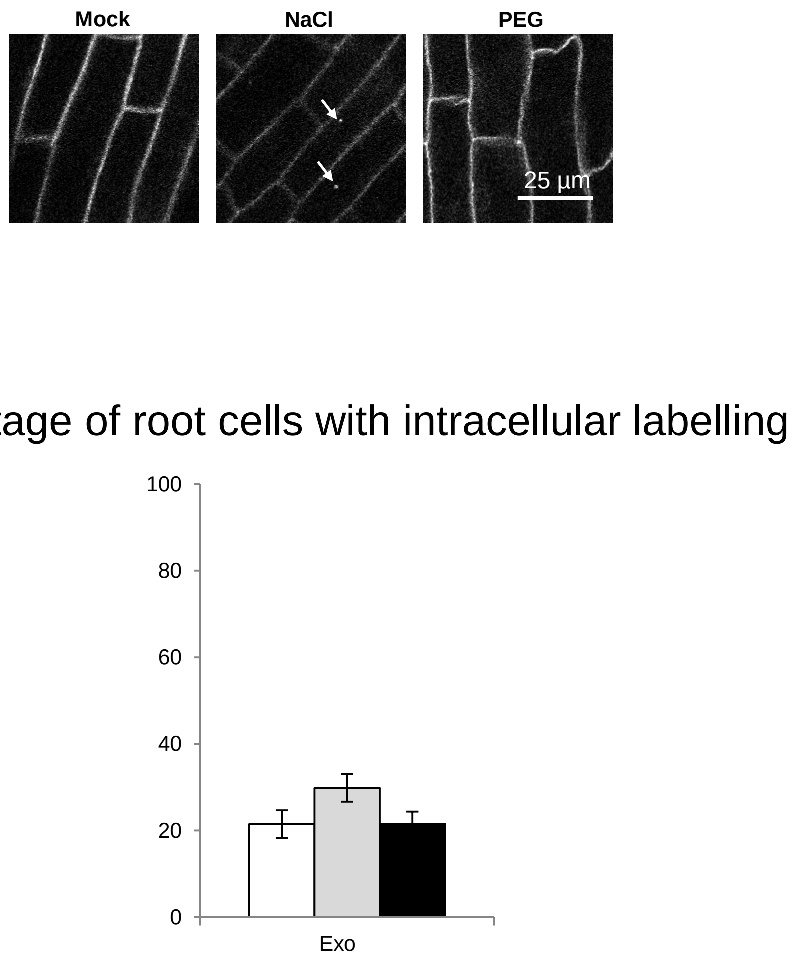

Supplementary Figure S5. Subcellular localization of plasma-membrane protein-marker LTi6a-CFP in rice root under salt and osmotic stresses

LTi6a-CFP construct was observed in crown roots. **(Upper)** Typical subcellular localization of LTi6a-CFP in exodermis observed by LSCM with intracellular labelling (pointed by arrows). **(Lower)** Charts show the percentage of root cells displaying intracellular labelling. Open, grey and closed bars correspond to mock (half-strength MS liquid medium), 100 mM NaCl and 20% (w/v) PEG6000, respectively. Experiments were done 2 times for each construct, 4-6 plants were observed for each experiment, ~10 cells were analysed for each plant. The mean values and SEMs are indicated.
