# Supplementary material for: Sub-cellular markers highlight intracellular dynamics of membrane proteins in response to abiotic treatments in rice
Source: Rice (N Y). 2018 Apr 12;11:23. doi: 10.1186/s12284-018-0209-2 (PMC5897272; doi:10.1186/s12284-018-0209-2)
Supplement: Supplementary file 2 — Table S1. Overview of molecular cloning of membrane protein markers. (DOCX 16 kb) [file 12284_2018_209_MOESM2_ESM.docx]

| Gene name | RAP locus ID | Origin of cDNA clone* | ID of cDNA clone | Forward and reverse primer sequences used to subclone cDNA fragment if PCR was applied | Cloning strategy** | Binary vector*** |
| --- | --- | --- | --- | --- | --- | --- |
| *OsPIP1;1* | Os02g0666200 | NIAS, Japan | 001-039-C05 | 5’-GGAGATAGAACCATGGAGGGGAAGGAGGAGGAC-3’  5’CAAGAAAGCTGGGTCTAAAGACCTGCTCTTGAAT3’ | G | pGWB5 |
| *OsPIP2;4* | Os07g0448100 | GenScript, USA | / | / | R | pGreen0179 |
| *OsPIP2;5* | Os07g0448400 | GenScript, USA | / | / | R | pGreen0179 |
| *OsTIP1;1* | Os03g0146100 | NIAS, Japan | 001-014-B06 | 5’-TCTCTCTCTAAGCTTATGCCGATCCGCAACATCGC-3’  5’-GCCACCACCTCCTAAGTAGTCGGTGGTGGGGAGCT-3’ | I | pGreen0179 |
| *OsTIP2;2* | Os06g0336200 | NIAS, Japan | J033044F19 | 5’TCTCTCTCTAAGCTTATGTCGGGCAACATCGCCTT-3’  5’GCCACCACCTCCTAAGAACTCGCTGCTGGCAACGG-3’ | I | pGreen0179 |
| *OsRab5a* | Os12g0631100 | NIAS, Japan | J013078I11 | 5’-GAGAAAGCTTATGGCGGCCAACCCCGGCAACAAGATCC-3’  5’-GACTCGAGCCACCACCTCCTGAGCAGCATGAAGAACTGCTC-3’ | R | pGreen0179 |
| *OsGAP1* | Os02g0709800 | NIAS, Japan | J013162A17 | 5’-TCTCTCTCTAAGCTTATGCGTCGAGAAGAAGCCTC-3’  5’-GCCACCACCTCCTAACATGACCTCGTCTTCTTGTA-3’ | I | pGreen0179 |
| *OsSCAMP1* | Os07g0564600 | NIAS, Japan | J033084E14 | 5’-TCTCTCTCTAAGCTTATGGCGGGGCGCTACGACAG-3’  5’-GCCACCACCTCCTAAAAAAGCTGCCCGCATAGCAC-3’ | I | pGreen0179 |

*NIAS: National Institute of Agrobiological Sciences. **G: Gateway® Gene Cloning (Invitrogen, USA), R: Restriction, I: In-Fusion® HD Cloning (Clontech, USA). ***pGWB5 (Nakagawa et al., 2007), pGreen0179 (Hellens et al., 2000).

**Table S1**
